# Relation between macular morphology and treatment frequency during twelve months with ranibizumab for diabetic macular edema

**DOI:** 10.1371/journal.pone.0175809

**Published:** 2017-04-13

**Authors:** Yuki Mori, Tomoaki Murakami, Kiyoshi Suzuma, Kenji Ishihara, Shin Yoshitake, Masahiro Fujimoto, Yoko Dodo, Tatsuya Yoshitake, Yuko Miwa, Akitaka Tsujikawa

**Affiliations:** Department of Ophthalmology and Visual Sciences, Kyoto University Graduate School of Medicine, Kyoto, Japan; International University of Health and Welfare, JAPAN

## Abstract

**Purpose:**

To investigate whether baseline optical coherence tomography (OCT) parameters can predict the treatment frequency of intravitreal ranibizumab (IVR) injections during the first year in patients with diabetic macular edema (DME) treated with pro re nata (PRN) IVR injections.

**Methods:**

We retrospectively reviewed 68 eyes of 63 patients with center-involved DME who received IVR injections for 12 months or longer according to three monthly IVR injections followed by the PRN dosing. We measured the mean retinal thicknesses in the individual subfields of the Early Treatment Diabetic Retinopathy Study grid and evaluated the qualitative and quantitative parameters on OCT sectional images. We investigated the relationship between these OCT parameters at baseline and the number of IVR injections during the 12-month follow-up.

**Results:**

Three loading doses were administered to 10 eyes; four to seven annualized IVR injections were administered to 34 eyes. The number of eyes that received IVR injections decreased gradually until month 6 and was almost constant from months 7 to 11. No relationships were seen between the treatment frequency and baseline systemic factors and the ophthalmic examination findings. Univariate analyses showed that the number of IVR injections during the first year was associated with the mean retinal thickness in the individual subfields and the transverse length of the disrupted external limiting membrane (ELM) and ellipsoid zone of the photoreceptors. Multivariate analysis showed a significant association with the thickness in the inferior subfield alone. The treatment frequency during the 12-month follow-up was not correlated with improved visual acuity but was associated with the decrease in the central subfield thickness and disrupted ELM.

**Conclusion:**

The retinal thickness in the inferior subfield predicts the treatment frequency during the first year in eyes with DME treated with PRN IVR injections.

## Introduction

Diabetic macular edema (DME), in which the blood-retinal barrier (BRB) is disrupted and neuroglial function deteriorates, often leads to visual impairment in patients with diabetes [1, 2]. Accumulated evidence in basic science and clinical studies has shown that vascular endothelial growth factor (VEGF) plays a key role in the molecular mechanisms in DME, which has encouraged ophthalmologists to treat DME with anti-VEGF drugs [3–7]. Two major drugs, ranibizumab (Lucentis, Novartis Pharma AG, Basel, Switzerland; and Genentech Inc., South San Francisco, CA, USA) and aflibercept (Eylea, Regeneron Pharmaceuticals, Tarrytown, NY, USA; and Bayer, Berlin, Germany) have been approved worldwide and are the first-line therapeutic strategy for managing DME [8, 9].

Considering the half-lives of these drugs in the vitreous humor, three major regimens, i.e., fixed monthly or bimonthly, pro re nata (PRN), and treat-and-extend (TAE), can be applied to treat DME [7–11]. A few clinical trials of intravitreal ranibizumab (IVR) injections for DME according to the PRN regimen have reported significant reductions in the treatment frequency in the second year and thereafter [12, 13]. Intriguingly, anti-VEGF therapy improves the severity of and retards progression of diabetic retinopathy (DR) and the expansion of the nonperfused areas in the macula [14, 15]. These data suggested that anti-VEGF drugs have rapid and direct effects on the disrupted BRB and exert slow and indirect mechanisms in DME resolution.

Despite the beneficial effects, the adverse effects of anti-VEGF management are rare but severe, e.g., endophthalmitis and life-threatening atherothrombotic diseases [16]. Clinical trials have suggested that anti-VEGF therapy guarantees the best improvement in visual acuity (VA), although the socioeconomic burden persists for patients. Actually, the cost-per-quality-adjusted life years is much higher than other conventional interventions for DME [17–19]. These issues might encourage clinicians to use the PRN regimen for DME and to optimize the indications for anti-VEGF therapy using predictors of visual prognosis and treatment frequency [20].

In the current study, we investigated the association of baseline systemic and ocular characteristics with the number of PRN IVR injections administered during the first year (3 + PRN regimen) to treat DME.

## Materials and methods

### Participants

We retrospectively reviewed 68 consecutive eyes of 63 patients with center-involved DME treated with IVR injections for 12 months or longer [21]. Patients with center-involved DME who visited Kyoto University Hospital from March 2014 to October 2015 as the baseline visit received three monthly injections followed by the PRN phase (3 + PRN regimen). Several patients dropped out during the 12-month follow-up because of inconvenience, patient desire to terminate treatment or change to other therapeutic strategies, drug tachyphylaxis, or additional treatments, i.e., focal/grid photocoagulation, panretinal photocoagulation, vitrectomy (for vitreous hemorrhage), or cataract surgery. All research and measurements adhered to the tenets of the Declaration of Helsinki. The Ethics Committee of the Kyoto University Graduate School and Faculty of Medicine approved the study protocol. All participants provided written informed consent before study enrollment.

### Intervention

Ranibizumab (0.5 mg) was administered intravitreally according to the 3 + PRN regimen described in the Ranibizumab Monotherapy or Combined with Laser versus Laser Monotherapy for Diabetic Macular Edema (RESTORE) study [8]. After disinfection, ranibizumab was injected 3.5 mm posterior to the limbus followed by instillation of antibiotics. Three monthly ranibizumab loading doses were followed by PRN IVR injections. At every monthly visit, the PRN treatment regimen was applied according to the retreatment criteria of the RESTORE study [8].

### Optical coherence tomography

After measurement of the VA and comprehensive ophthalmic examinations, we acquired sectional and three-dimensional images using spectral-domain optical coherence tomography (SD-OCT) (Spectralis OCT, Heidelberg Engineering, Heidelberg, Germany) at every monthly visit. After calibration using the corneal curvature radii and focusing knob, vertical and horizontal sectional images dissecting the fovea were acquired using the cross-hair mode (30 degrees). Three-dimensional images were obtained according to the following parameters; a 49 line raster scan (central 20 x 20 degree), automatic real-time mean of 16, and high resolution (512 A-scans/B-scan). Two-dimensional maps subsequently were constructed using the manufacturer’s software. The technology enabled automatic measurement of the mean retinal thicknesses of the central subfield (CSF) and the individual subfields in the parafovea of the Early Treatment Diabetic Retinopathy Study (ETDRS) grid, as described previously [22, 23].

We evaluated qualitatively and quantitatively several OCT parameters at the fovea. Briefly, we determined the presence or absence of cystoid macular edema (CME) and serous retinal detachments (SRD) and measured the height of the foveal cystoid spaces or SRD at the presumed foveal center in eyes with CME or SRD using the caliper tool in the Heidelberg Eye Explorer software (Heidelberg Engineering) [23]. Vitreomacular traction (VMT) was defined as the presence of an epiretinal membrane involving the fovea or posterior vitreous membrane with foveal traction on the vertical and horizontal retinal sections of the SD-OCT images. Vitreomacular adhesion (VMA) was defined as attachment of the posterior vitreous cortex according to the modified methods described recently [24]. We evaluated the elevation of the perifoveal vitreous cortex from the retinal surface along with attachment of the vitreous cortex at the foveal center on the vertical and horizontal sectional OCT images. Such eyes with or without VMT were included in the group with VMA in this study.

We measured the transverse length of the disrupted ellipsoid zone of the photoreceptors (EZ) or external limiting membrane (ELM) on the vertical and horizontal images dissecting the fovea as reported previously [25]. Briefly, we first excluded the areas where reflectivity signals in the retinal pigment epithelium were attenuated by medial opacity or hyperreflective lesions in the inner retinal layers, and the areas with damaged photoreceptors then were quantified. The status of the EZ lines was divided into three categories, i.e., intact, faint, and disrupted, according to the OCT reflectivity. The ELM status was defined as intact or disrupted, because the reflectivity levels in the ELM were almost constant. We thus quantified the transverse length of the areas where the EZ or ELM line was disrupted within the central 1 mm on the vertical and horizontal images using the caliper tool in the Heidelberg Eye Explorer software. The average of the percentage was used in the subsequent investigations. Disorganization of the inner retinal layers also was measured as the transverse length of the central areas where the inner retinal layers could not be segmented clearly as reported previously [26]. Two independent retinal specialists evaluated these OCT parameters. If there was disagreement regarding the qualitative parameters, a third specialist participated. For the quantitative parameters, the average of the values obtained by two specialists was applied to further analyses.

### Statistical analysis

The results are expressed as the median (interquartile range). The differences between two groups were evaluated using the Wilcoxon signed-rank test or the Mann-Whitney *U*-test. Spearman’s correlation coefficient was calculated to test the statistical correlation. For the multivariate analysis, we performed multiple regression analysis using a stepwise forward approach (the mean retinal thicknesses in the CSF and the individual parafoveal subfields [nasal, temporal, superior, and inferior] and the transverse length of the disrupted ELM and EZ as independent variables and the number of IVR injections during the 12-month follow-up as a dependent variable). *P* < 0.05 was considered significant.

## Results

### Association of baseline characteristics with the number of IVR injections during the first 12 months

We retrospectively reviewed 68 eyes of 63 patients with center-involved DME who received PRN IVR injections during the first 12 months. Among 125 eyes that met the eligibility criteria, nine eyes met the exclusion criteria at baseline. Of the remaining 116 eyes, 48 eyes were lost to follow-up before the 12-month examination. Table 1 shows the baseline systemic and ocular characteristics.

**Table 1:** Baseline characteristics.

Parameter	
Eyes/patients	68/63
Mean age	69 years (range, 59–74)
Gender Men Women	35 28
Hemoglobin A1c	7.2% (range, 6.6–7.8)
Systemic hypertension Absent Present	26 37
LogMAR VA	0.260 (0.155–0.506)
Lens status Phakia Pseudophakia	45 eyes 23 eyes
DR severity Mild NPDR Moderate NPDR Severe NPDR PDR	1 eye 37 eyes 13 eyes 17 eyes
Previous PRP Absent Present	25 eyes 43 eyes

HbA1c: hemoglobin A1c; logMAR: logarithm of the minimum angle of resolution; NPDR: nonproliferative diabetic retinopathy; PDR: proliferative diabetic retinopathy; PRP: panretinal photocoagulation.

**Fig 1:**
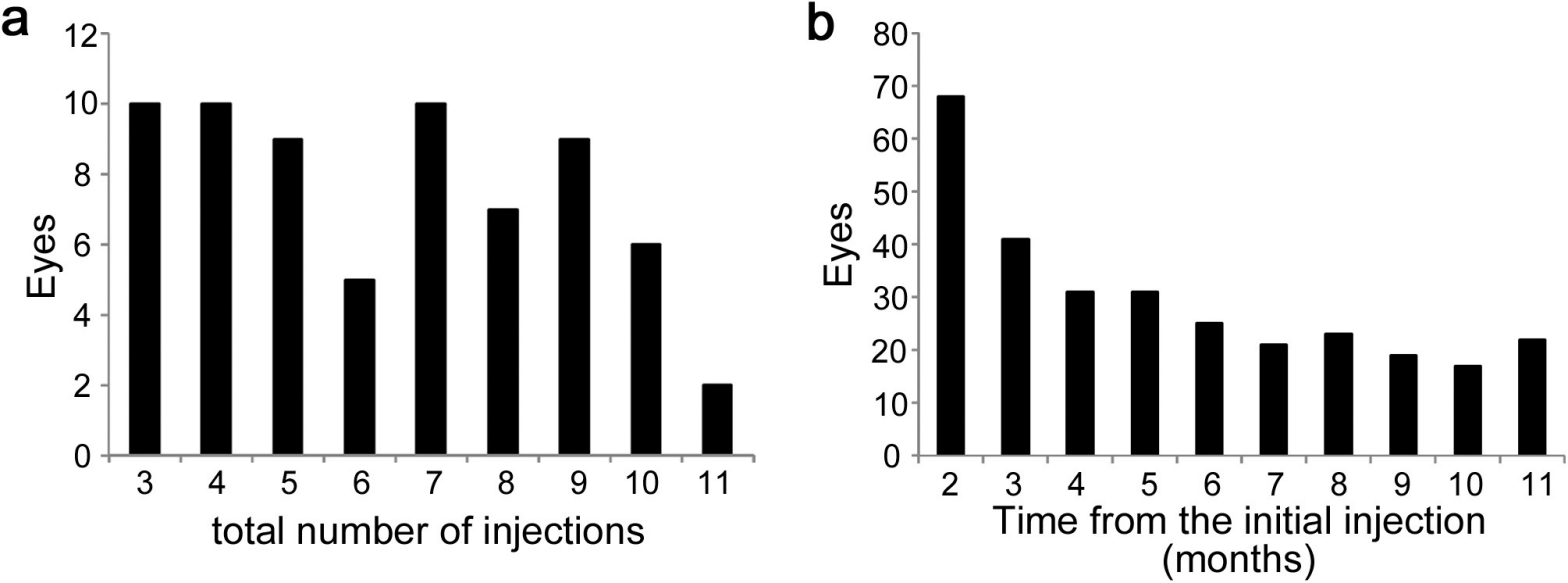
The number of IVR injections during the first year. (**a**) Individual numbers of IVR injections during the 12-month follow-up. (**b**) IVR injections at individual time points.

All 68 eyes received PRN IVR injections during the 12-month follow-up; only 10 eyes received three loading doses alone (Fig 1A). Thirty-four eyes received four to seven annualized injections, and 24 eyes required eight or more IVR injections. We further investigated the number of eyes that received IVR injections at individual time points. IVR injections were administered to 41 eyes at month 3 and the number of eyes decreased gradually until month 6 (Fig 1B). The number of eyes that received IVR injections was almost constant from months 7 to 11.

We evaluated the relationship between the baseline characteristics and treatment frequency of IVR injections during the 12-month follow-up and did not identify any significant associations with systemic factors or the findings on ophthalmic examination (Table 2).

**Table 2:** Relationship between preoperative parameters and the number of IVR injections during the 12-month follow-up.

Baseline parameter	Association with number of IVR injections
Age	*ρ* = -0.206, *p* = 0.091
Gender Men Women	*p* = 0.350 7 (5–9) 5 (4–8)
Hemoglobin A1c	*ρ* = -0.217, *p* = 0.117
Systemic hypertension Absent Present	*p* = 0.440 5 (4–8) 7 (4.75–9)
LogMAR VA	*ρ* = 0.126, *p* = 0.301
Lens status Phakia Pseudophakia	*p* = 0.399 6 (4–8) 7 (4.5–9)
DR severity Mild NPDR Moderate NPDR Severe NPDR PDR	5 6 (4–8) 8 (4–9) 7 (4–9)
Previous PRP Absent Present	*p* = 0.871 7 (4–8) 6 (4–9)

logMAR: logarithm of the minimum angle of resolution; NPDR: nonproliferative diabetic retinopathy; PDR: proliferative diabetic retinopathy; PRP: panretinal photocoagulation.

The *p-*value indicates Spearman’s correlation coefficient for the association of the number of IVR injections with age, HbA1c, and logMAR VA. The Mann-Whitney *U*-test was performed to compare gender, systemic hypertension, lens status, and previous panretinal photocoagulation.

The investigation using OCT parameters showed a positive association between the CSF thickness and the number of IVR injections during the first year (*ρ* = 0.408, *p*<0.001) (Table 3). Intriguingly, the treatment frequency also was related to the mean retinal thicknesses in the individual subfields of the parafovea. Among them, the retinal thickness in the inferior subfield had the most significant association with the number of IVR (*ρ* = 0.516, *p*<0.001) (Table 3).

**Table 3:** Association between retinal thickness and number of IVR injections during the 12-month follow-up.

Subfield	Thickness	Association with number of IVR injections
Fovea	441 μm (402–549)	*ρ* = 0.408, *p*<0.001
Parafovea		
Nasal	408 μm (372–468)	*ρ* = 0.483, *p*<0.001
Temporal	470 μm (389–549)	*ρ* = 0.382, *p* = 0.002
Superior	433 μm (389–519)	*ρ* = 0.277, *p* = 0.023
Inferior	414 μm (362–503)	*ρ* = 0.516, *p*<0.001

The *p*-value indicates Spearman’s correlation coefficient for the association between the number of IVR injections and the mean retinal thicknesses in the individual subfields.

Several OCT parameters other than the retinal thicknesses also were related to the visual outcomes after treatment with anti-VEGF drugs for DME, which encouraged us to investigate their association with the number of injections [27–29]. The number of IVR injections in eyes with CME or SRD did not differ from the number in eyes without such findings (Table 4). The treatment frequency was related to the height of the foveal cystoid spaces in 54 eyes with CME (*ρ* = 0.431, *p* = 0.002) but not to the height of the SRD in 21 eyes (*ρ* = 0.339, *p* = 0.130). The disruption of the ELM line or EZ line, which represents photoreceptor damage, was associated positively with the number of IVR injections (Table 4). Multivariate analyses showed that the treatment frequency was related to the retinal thickness in the inferior subfield alone (*β* = 0.492, *p*<0.001) among these baseline characteristics.

**Table 4:** Relationship between other OCT parameters and the number of IVR injections during the first year.

OCT parameter at baseline		Relation to number of IVR injections
CME Absent Present	14 eyes 54 eyes	*p* = 0.446 7 (4.25–9) 6.5 (4–8)
SRD Absent Present	47 eyes 21 eyes	*p* = 0.304 6 (4–8) 8 (4–9)
VMT Absent Present	64 eyes 4 eyes	*p* = 0.360 6 (4–8.25) 8.5 (6.75–9.25)
VMA Absent Present	45 eyes 23 eyes	*p* = 0.952 6 (4–9) 7 (5–8)
Transverse length of disrupted ELM	0% (0–4.4)	*ρ* = 0.377, *p* = 0.002
Transverse length of disrupted EZ	9.6% (0–27.2)	*ρ* = 0.262, *p* = 0.032
DRIL	60.8% (36.8–84.5)	*ρ* = -0.001, *p* = 0.991

The *p-*value indicates the Mann-Whitney *U*-test for the comparison of CME, SRD, VMT, and VMA. Spearman’s correlation coefficient indicates the association of the number of IVR injections with the transverse length of the disrupted ELM or EZ and DRIL.

DRIL, disorganization of the inner retinal layers.

### Relationship between treatment frequency and functional or morphologic outcomes

The logMAR VA and CSF thickness improved significantly at 12 months (*p*<0.001 for both comparisons) (Tables 1, 3 and 5). The mean retinal thicknesses in the parafovea were also improved, and there were no differences in the changes of the retinal thicknesses between individual subfields of the parafovea. There was no association between the number of IVR injections and the visual outcomes or visual improvement (Table 5). The number of IVR injections during the first year was unrelated to the CSF thickness per se but to its decrease at month 12 (*ρ* = 0.120, *p* = 0.370 and *ρ* = 0.328, *p* = 0.007, respectively) (Table 5). The treatment frequency also tended to be associated positively with the decrease in the transverse length of the disrupted EZ or ELM line (*ρ* = 0.212, *p* = 0.083 and *ρ* = 0.287, *p* = 0.019, respectively) (Table 5).

**Table 5:** Relation between visual outcomes at 12 months and number of IVR injections during the 12-month follow-up.

Parameters at 12 months		Relation to number of IVR injections
LogMAR VA	0.155 (0.046–0.301)	*ρ* = 0.004, *p* = 0.971
VA improvement	0.125 (0.030–0.234)	*ρ* = 0.177, *p* = 0.146
CSF thickness	314 μm (269–386)	*ρ* = 0.120, *p* = 0.370
Decrease in CSF thickness	132 μm (54–191)	*ρ* = 0.328, *p* = 0.007
Nasal thickness	356 μm (338–389)	*ρ* = 0.240, *p* = 0.049
Decrease in nasal thickness	45 μm (13–94)	*ρ* = 0.420, *p*<0.001
Temporal thickness	371 μm (341–392)	*ρ* = 0.172, *p* = 0.162
Decrease in temporal thickness	74 μm (32–180)	*ρ* = 0.323, *p* = 0.009
Superior thickness	365 μm (344–390)	*ρ* = 0.300, *p* = 0.013
Decrease in superior thickness	61 μm (22–139)	*ρ* = 0.205, *p* = 0.102
Inferior thickness	352 μm (333–382)	*ρ* = 0.230, *p* = 0.059
Decrease in inferior thickness	59 μm (17–126)	*ρ* = 0.522, *p*<0.001
Transverse length of disrupted EZ	0% (0–9.0)	*ρ* = 0.294, *p* = 0.016
Decrease in disrupted EZ	4.1% (0–17.2)	*ρ* = 0.212, *p* = 0.083
Transverse length of disrupted ELM	0% (0–0)	*ρ* = 0.405, *p*<0.001
Decrease of disrupted ELM	0% (0–0)	*ρ* = 0.287, *p* = 0.019

The *p* value indicates the Spearman’s correlation coefficient for the association between the number of IVR injections and individual parameters.

## Discussion

Anti-VEGF treatment improves both visual function and macular morphology in DME, whereas the severe complications and socioeconomic burden suggest the need to predict the treatment frequency on a PRN dosing schedule [8, 16–19]. In the current study, we showed for the first time that the number of IVR injections during the first year was associated positively with the retinal thickness in the inferior subfield of the ETDRS grid in eyes that received IVR injections according to the 3 + PRN regimen. The data are feasible for use in patient consultations in the clinic and to reduce the treatment burden of the health care system.

Several clinical trials have reported significant reductions in the treatment frequency with IVR injections during the second year and afterward in eyes treated with PRN IVR injections for DME [12, 13]. These publications have suggested that the burden of the anti-VEGF treatment depends substantially on the treatment frequency during the first year and, concomitantly, predicting the treatment frequency during this period is an important issue in anti-VEGF treatment. The current study showed an association between the baseline retinal thickness and the number of IVR injections during the first year. It might to some extent be consistent with the findings of a recent study that showed that, among several parameters, the baseline central foveal thickness predicts the number of IVR injections needed during the PRN open-label extension IVR periods after 3-year monthly IVR injections [20].

In the current study, the participants received IVR injections alone during the 12-month follow-up. The major rescue protocol is macular photocoagulation and might reduce the frequency of IVR treatment [30]. Other publications have reported fewer injections of anti-VEGF drugs combined with triamcinolone, despite no differences in the visual outcomes [31]. Although there were no differences in the treatment frequency between eyes with and without VMT in the current study, most clinicians believe that VMT often prevents anti-VEGF treatment from achieving complete resolution of DME [32]. Other publications have reported the efficacy of IVR injections in vitrectomized eyes, which suggests that vitrectomy followed by anti-VEGF therapy might be a possible alternative strategy and reduce the treatment frequency in eyes with both DME and VMT [33, 34].

The association between the retinal thickness and treatment frequency with IVR injections suggests that the magnitude of vascular hyperpermeability might be related to the need for more frequent injections and might be consistent with the relationship between fluorescein leakage and the number of injections in a recent publication [20]. VEGF induces vascular hyperpermeability via several mechanisms. VEGF increases the paracellular or transcellular flux in vascular endothelial cells [5, 35] and inhibits pericyte function and disrupts the endothelial-pericyte interaction, which contributes to both angiogenesis and vascular permeability [36]. VEGF-induced expression of intercellular adhesion molecule-1 contributes to leukostasis [37]. Concomitantly, retinal capillaries are occluded transiently, and extravasated inflammatory cells increase cytokines and vascular permeability [4, 38, 39]. The direct effects of anti-VEGF drugs on vascular endothelial cells is rapid and transient. In contrast, the indirect mechanisms via negative regulation of pericyte function, transient capillary nonperfusion, or infiltrated inflammatory cells might be slowly exacerbated or improved and might require prolonged and repeated administration of anti-VEGF drugs [14, 15, 40, 41].

In the current study, the retinal thickness in the inferior subfield had a more significant association with the treatment frequency with IVR injections than the CSF thickness. The baseline height of the foveal cystoid spaces also was correlated with the treatment frequency. Although the implications of retinal thickening in the inferior subfield in DME remain unknown, we hypothesized that persistent and prolonged edematous changes might impair the mechanical integrity, which would allow the extracellular fluid to migrate toward the inferior subfield as a result of gravity. In other words, retinal thickening in the inferior subfield might represent old DME to some extent. A recent study reported an association between the height of the foveal cystoid spaces and the retinal thickness in the inferior subfield in DME [23]. Eyes of CME type often have an enlarged foveal avascular zone, which suggests older DME [42]. Unfortunately, since most patients in the current study were referred to our institution, we did not know definitively the duration of diabetes, DR, or DME before the initial injection. A future study should determine whether persistent DME is associated with larger foveal cystoid spaces and retinal thickening in the inferior subfield and needs more frequent treatment with ranibizumab.

Univariate analyses showed the association of the treatment frequency with the disrupted ELM at baseline, although multivariate analysis revealed that the number of IVR injections was related to the retinal thickness in the inferior subfield alone. Statistical analysis suggested that the disrupted ELM was a confounding factor, because there was the association between the retinal thickness and the disrupted ELM (data not shown). It is consistent with the shadow artifacts that the ELM lines are often disrupted beneath severe retinal edema. In addition, further investigation should be planned to elucidate whether the treatment frequency was related to the photoreceptor status in the individual subfield.

The number of IVR injections was associated with improved retinal thickening and photoreceptor damage but not with VA improvement at month 12. The absence of an association between functional and anatomic responses to repeated IVR injections suggested the presence of other mechanisms in visual impairment than edematous changes, which is consistent with the modest correlation between CSF thickness and VA reduction reported by the Diabetic Retinopathy Clinical Research Network [43]. In addition, we wonder if we can identify the eyes with greater VA improvement after fewer IVR injections. Further studies should reveal novel mechanisms independent of edematous changes as novel therapeutic targets [44].

The current retrospective study with a smaller number of Asian cases had several limitations. Another study should determine if these results are generalizable to longer periods, other populations, and other regimens, e.g., the TAE regimen and the PRN regimens with different loading doses. Since we excluded patients who received additional treatment in the current study, eyes less responsive to anti-VEGF drug might have been omitted from this study. Anti-VEGF drugs affect vascular cells, and the predictors on fluorescein angiography or OCT angiography images should be reported in a future study [45].

## Conclusions

The current study designated baseline macular thickness as a predictor of the treatment frequency during the first year in eyes that followed PRN IVR injection regimen to treat DME, suggesting its feasibility in the clinic and regarding socioeconomic issues.
